# A double‐edged sword: The complex interplay between engineered nanoparticles and platelets

**DOI:** 10.1002/btm2.10669

**Published:** 2024-04-06

**Authors:** Yathreb Asaad, Danielle Nemcovsky‐Amar, Josué Sznitman, Pierre H. Mangin, Netanel Korin

**Affiliations:** ^1^ Department of Biomedical Engineering Technion‐Israel Institute of Technology Haifa Israel; ^2^ University of Strasbourg, INSERM, EFS Grand‐Est, BPPS UMR‐S1255, FMTS Strasbourg France

**Keywords:** drug carriers, hemostasis, nanoparticles, physicochemical parameters, platelets, systemic delivery, thrombosis

## Abstract

Nanoparticles (NP) play a crucial role in nanomedicine, serving as carriers for localized therapeutics to allow for precise drug delivery to specific disease sites and conditions. When injected systemically, NP can directly interact with various blood cell types, most critically with circulating platelets. Hence, the potential activation/inhibition of platelets following NP exposure must be evaluated a priori due to possible debilitating outcomes. In recent years, various studies have helped resolve the physicochemical parameters that influence platelet‐NP interactions, and either emphasize nanoparticles' therapeutic role such as to augment hemostasis or to inhibit thrombus formation, or conversely map their potential undesired side effects upon injection. In the present review, we discuss some of the main effects of several key NP types including polymeric, ceramic, silica, dendrimers and metallic NPs on platelets, with a focus on the physicochemical parameters that can dictate these effects and modulate the therapeutic potential of the NP. Despite the scientific and clinical significance of understanding Platelet‐NP interactions, there is a significant knowledge gap in the field and a critical need for further investigation. Moreover, improved guidelines and research methodologies need to be developed and implemented. Our outlook includes the use of biomimetic in vitro models to investigate these complex interactions under both healthy physiological and disease conditions.

**Translational Impact Statements**

Understanding how nanoparticles interact with platelets and their potential effects on health and disease conditions is critical for the design of safe and effective therapies that can be translated into clinical practice.

## 1 INTRODUCTION

The last decade has witnessed an innovative revolution in the field of nanomedicine, leveraging the fabrication of nanoparticles (NPs) for miscellaneous medical applications. NPs, which range from 1 to 100 nm in size, hold promising potentials for a plethora of pathological diagnosis, drug delivery, disease prevention and even therapy. Such potentials can be applied in various applications, such as cellular and molecular imaging and tissue engineering. Moreover, NPs can offer distinctive advantages that can overcome important pending issues in terms of drug solubility, biodistribution, pharmacokinetics and pharmacodynamics.^1^ Among their recognized benefits, the high surface area of NPs endows them desirable characteristics in terms of penetrating biological tissues and high delivery and loading concentrations at targeted sites. However, upon injection into the bloodstream, this specific characteristic can also lead to undesirable side effects due to the high interaction potential between NPs and other blood cells, including erythrocytes, white blood cells and platelets.^2^, ^3^ Within these cell‐particle interactions, understanding the interactions with platelets is fundamental to advancing injectable NP‐based technologies as these may result in debilitating outcomes.

**FIGURE 1:**
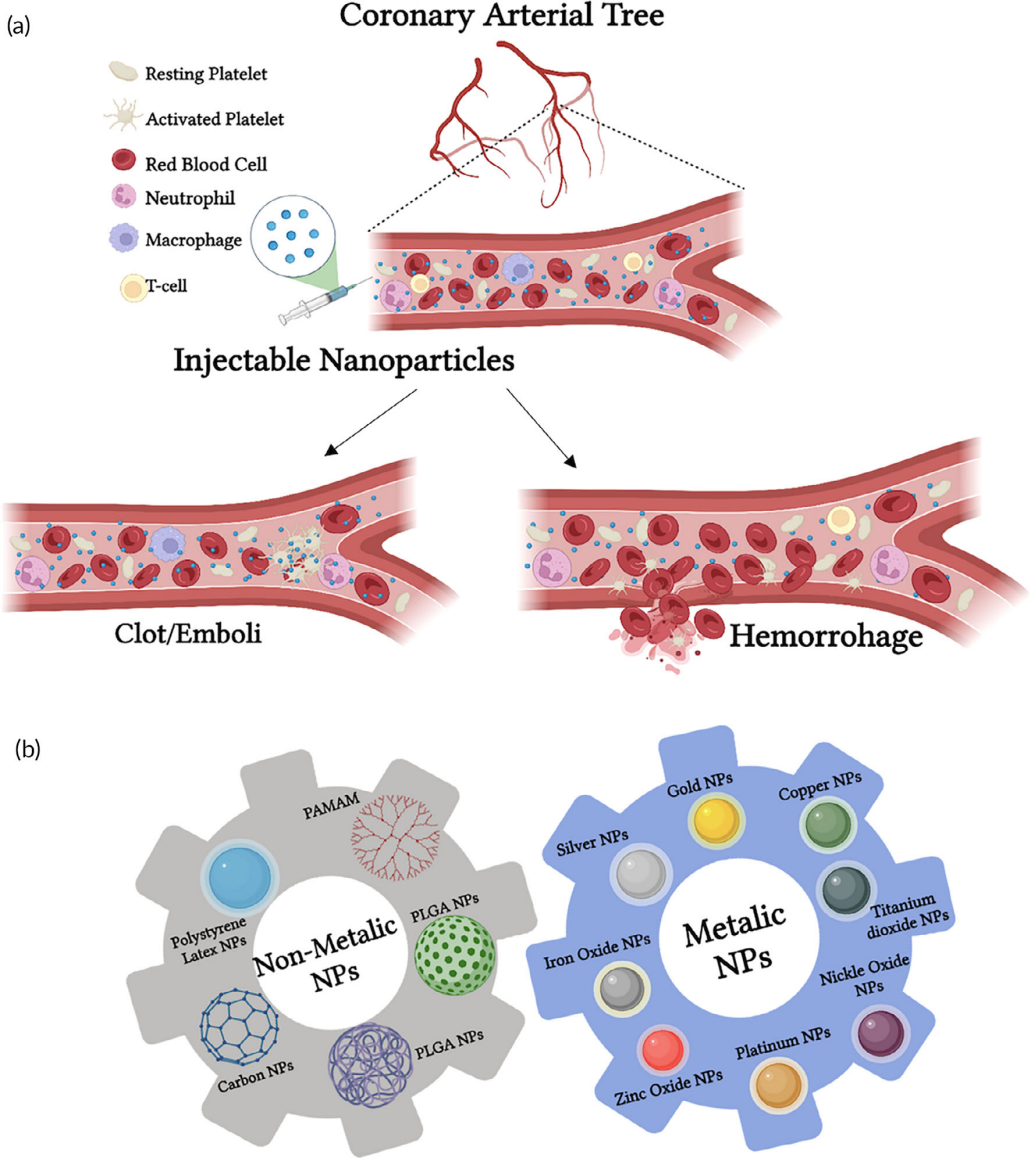
Upon injection into the bloodstream, different NPs can affect differently circulating platelets. (a) NPs possess high surface area and thus have high interaction potential with circulating blood cells. This can also result in undesirable side effects such as thrombotic or bleeding complications. (b) Summary of the different types of engineered NPs, that is, polymeric, ceramic, silica, dendrimers, and metallic NPs covered in this review. Created with BioRender.com.

Briefly, platelets are a nuclear specialized blood cell that play a pivotal role in maintaining hemostasias, inflammation, tumor metastasis, wound healing, and host defense. Material based contact between platelets and particles can lead to hemo‐incompatibility. This trigger can activate platelets, via several mechanisms, and initiate a thrombotic response. The activated platelets can induce and escalate thrombosis by several pathways leading to clot formation at undesired sites; the latter can lead to deadly outcomes. Therefore, mal‐regulation or dysfunction in platelets can deviate our body from its hemostatic equilibrium and result in pathological medical conditions, such as hemorrhagic and thrombotic disorders^4^, ^5^, ^6^ (see Figure 1a).

Among the possible side effects that injectable particles can induce, thrombosis is likely recognized as the most life‐threatening event. There are two main types of thrombosis: arterial and venous thrombosis. Arterial thrombosis is a blood clot formed within an artery under high dynamic rheological condition, whereas venous thrombosis is a blood clot within a vein under low shearing forces.^7^ Platelets play the maestro role in orchestrating arterial thrombosis while in venous thrombosis, their role is believed to be peripheral. Although discussing platelets' role in arterial and venous thrombosis is not within the central scope of this review, mentioning platelets' role in venous thrombosis can delineate the potential effects of NPs on platelets more precisely. Recent studies show that arterial and venous thrombosis dichotomy are not as simple as always referred to.^8^, ^9^ In fact, both can be associated with and linked to specific clinical conditions. Although arterial thrombosis bestows the lion's share in evaluating NPs compatibility on platelets, as it can lead to deadly myocardial infraction and stroke, venous thrombosis also needs to be carefully evaluated with more future studies to delineate unknown risks.

**FIGURE 2:**
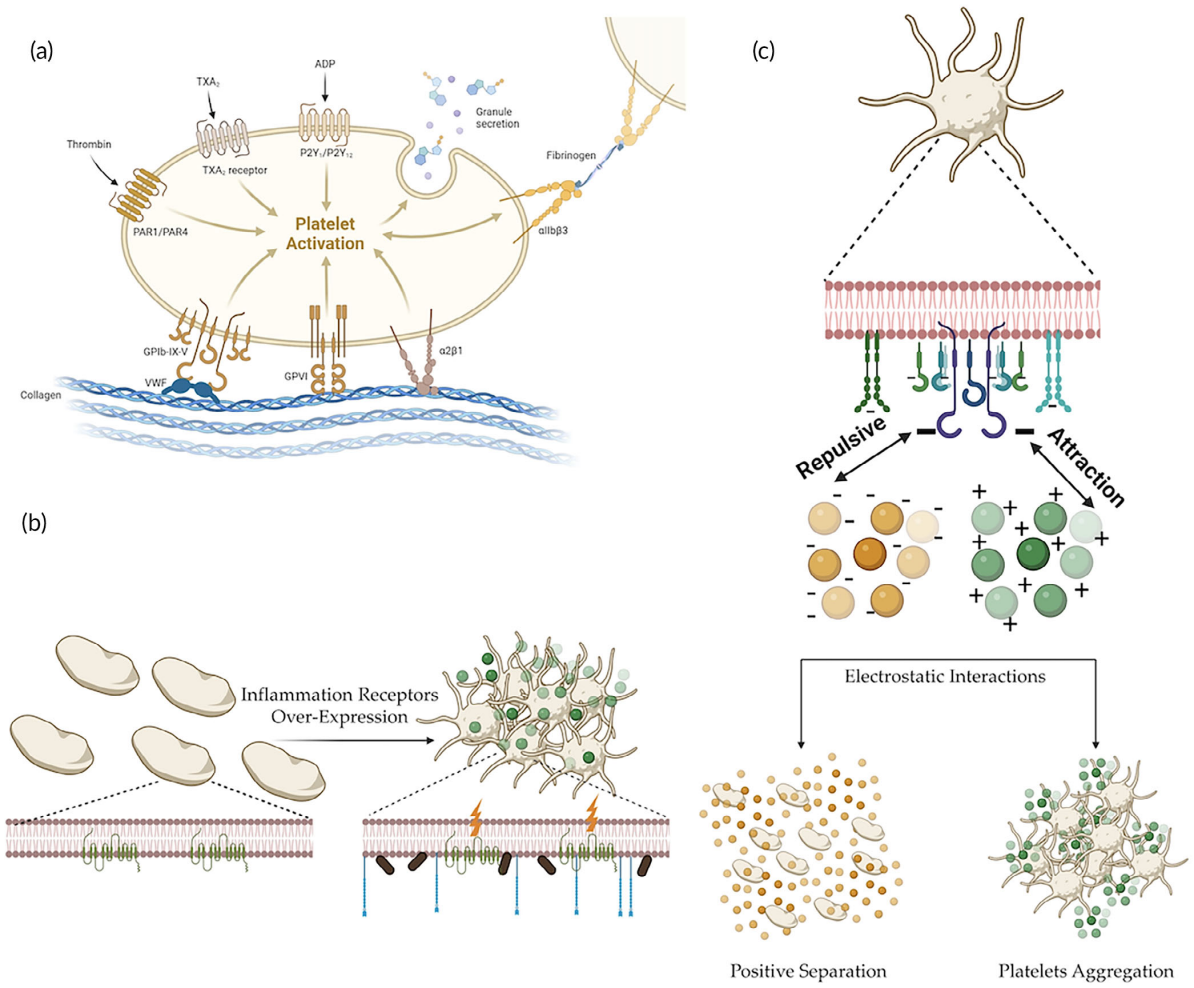
NP‐induced changes in platelet function: direct protein‐receptor activation, indirect activation, and electrostatic interactions. (a). Platelets can be directly activated via several pathways and protein‐receptor mediators. (b) Platelets can be activated by reciprocal interactions with particles which are indirect. Receptors such as PAC‐1, CD62‐P and Annexin‐V are over‐expressed in several cases. (c) A platelet's surface is negatively charged. Generally summarized, particles can either promote thrombus formation or not interfere, due to attractive or repulsive electrostatic interactions, respectively. Created with BioRender.com.

The reciprocal interactions between platelets and NPs are a strong function of various parameters. Various studies have demonstrated that physiochemical properties of particles such as matrix material, shape, size, surface chemistry, surface charge and concentration affect platelet function.^10^ For example, the physical contact of particles with platelets can lead to their activation via protein‐receptor interactions, see Figure 2a. Platelet activation can also result from indirect interactions mediated by other cells and factors, see Figure 2b. Platelet surfaces are negatively charged, so positively charged NPs can electrostatically attach to negatively charged surfaces, inducing platelet aggregation and activation (e.g., ATP release), see Figure 2c. However, negatively charged NPs can sometimes also separate platelets apart and inhibit thrombosis, as will be explained later. Moreover, there are a variety of other mechanisms that dictate platelet‐NP interactions, where the exact mechanism that drives these effects is still to be resolved. In this context, there is a critical need to investigate the reciprocal interactions between platelets and NPs to decipher any potential effects on platelet function.

The subject of nanoparticle (NP) interaction with platelets and blood is multifaceted and affects various aspects of nanomedicine, including NP biodistribution, toxicity, and blood clearance.^11^ In this review, we focus on NP‐platelet interactions addressing different NP types and their effects on platelet function, hemostasis, and thrombosis. We also highlight the methods used for such studies. We provide an overview of the primary interactions of several types of NPs (i.e., polymeric, ceramic, silica, dendrimers, and metallic NPs; see Figure 1b and Table 1), which exhibit distinctive physicochemical characteristics. Specifically, we discuss the effects of size, shape, charge, and matrix on platelet function. Finally, we briefly emphasize the need for further research on these interactions and the importance of investigating them in realistic health and disease conditions using advanced biomimetic in vitro models.

**TABLE 1:** Different types of nonmetallic and metallic nanoparticles characteristics and application.

Type of material	Type of NP	Characteristics and applications
Nonmetallic	Polystyrene latex NPs	Broad size range, hydrophobic surface, monodispersed, wide range of fluorescent colors^12^
	PLGA NPs	Biocompatible, biodegradable, surface can be easily modified^23^
	Carbon NPs	Unique physiochemical properties, bioimaging use^29^
	Silica NPs	High thermal and chemical stability, biocompatible^3^
	PAMAM dendrimers	Polymeric branches with core^42^
Metallic	Gold NPs	Targeted thermal therapy and imaging^52^
	Silver NPs	Antimicrobial properties^56^
	Iron oxide NPs	Paramagnetic features^50^
	Zinc Oxide NPs	FDA‐approved metal oxide for chemotherapy, drug and gene delivery^68^
	Nickel oxide NPs	Mostly used for energy, such as fuel cell^72^
	Copper NPs	Sensitive to oxygen and water. Antimicrobial and antibacterial properties^73^
	Titanium dioxide NPs	Photoactive^75^
	platinum NPs	Intrinsic catalytic properties^77^

## 2 NON‐METALIC NPS

### 2.1 Polystyrene (PS) latex NPs

Latex particles are polymer particles, in the size range of 1 to 1000 nm, formed from an amorphous polymer such as polystyrene (PS). PS particles possess a hydrophobic surface that can physically adsorb various biological molecules through passive adsorption.^12^ However, several chemical surface modifications can be applied to alter surface characteristics and expand their applications. Furthermore, PS particles exhibit numerous exceptional characteristics, including monodispersity, a wide range of particle sizes, fluorescent colors, and widespread commercial availability, making them a popular choice for basic studies. The hemocompatibility of PS particles has been extensively investigated in the literature, with numerous studies dedicated to elucidating the effects of PS particles on blood components, especially platelets.

In the seminal study of 2002, Nemmar et al.^13^ established and validated an injury model in hamsters, in which mild damage was photochemically induced to endothelial cells, leading to platelet‐rich thrombus formation. In their study, 60 nm PS NP of different zeta potential (anionic, cationic and unmodified) were intravascularly administrated. The results showed that unmodified PS particles did not interfere with thrombus formation, even at a high dose of 5000 μg/kg. In contrast, negatively charged particles inhibited thrombus growth at 100 μg/kg, while positively charged amine‐modified PS NP enhanced thrombosis at 5 μg/ml. The precise mechanism behind these findings is still speculated, with electrostatic interactions between platelet surfaces and amine particles suggested as a key factor. As stated earlier, the amine‐modified particles induced thrombosis in vivo which is consistent with the fact that these particles increased the ADP‐triggered platelet aggregation. Another study by Eun‐Hye et al.^14^ found that amine modified PS NPs increased thrombus formation via RBCs activation. For a more extensive understanding of this observation, the researchers suggested that electrostatic interactions between platelets' surface and amine particles were the key to this enigma.^13^ As mentioned, platelets enjoy a net negative charge on their surface due to ionizable sialic acid groups.^15^ Positively charged amine particles are presumed to interact with these sialic acid groups through electrostatic interactions, bridging between platelets and explaining the observed large aggregates under microscopic examination. This theory aligns with a previous finding by Taketomi and Kuramoto,^16^ where cationic poly‐peptide poly‐lysine induced platelet aggregation when added to plasma‐rich platelets. In contrast, negatively charged carboxylated particles exhibited an inconsistency in behavior, down‐regulating thrombosis in vivo while weakly enhancing ADP‐induced platelet aggregation in vitro.

Concurrently, McGuinnes et al.^17^ explored the effect of PS NPs of three distinctive surface chemistries (i.e., anionic, cationic, and unmodified) for a fixed size of 50 nm on citrated whole blood (11 mM sodium citrate) and isolated platelets in vitro. When added to whole blood and incubated, all examined NPs promoted platelet‐monocyte aggregation. However, they differentially promoted platelet–platelet aggregation. Unmodified PS particles did not induce platelet–platelet aggregation, while both cationic and anionic particles promoted thrombus formation. This suggests that platelet‐monocyte binding does not necessarily predict thrombus formation caused by direct interaction with particles. To understand the mechanisms behind cationic and anionic PS NPs promoting thrombus formation, the study measured the expression of PAC‐1, CD62‐P, and Annexin V following platelet exposure to the three particle types. Cationic PS particles significantly increased the expression of CD62P and PAC‐1, while both anionic and unmodified particles did not. Furthermore, both anionic and cationic PS NPs induced significant Annexin V binding, indicating the relocation of phosphatidylserine to the outer plasma membrane.^17^ This latter study highlights the importance of surface properties in inducing platelet aggregation, determining the mechanism by which the aggregation cascade is initiated. Anionic PS particles appears to upregulate adhesion receptors, while cationic PS NPs act via an unexplained mechanism by displaying anionic phospholipids on the external surface of the platelet plasma membrane.^17^

In yet another study investigating the effect of charge and size on platelets, Smyth et al.^18^ used PS NPs (i.e., cationic, anionic, and neutral NPs) with a diameter of 50 and 100 nm. All examined PS NPs induced apparent concentration‐dependent (15–60 μg/ml) platelet aggregation as shown as increase in light transmission measurements in isolated platelet suspensions. For the 50 nm PS NPs tested, the carboxylated NPs were the most potent, while the unmodified NPs were the least. In contrast, for the 100 nm PS NPs the opposite results were obtained where the unmodified NPs were the most potent whereas carboxylated NPs were the least.

**FIGURE 3:**
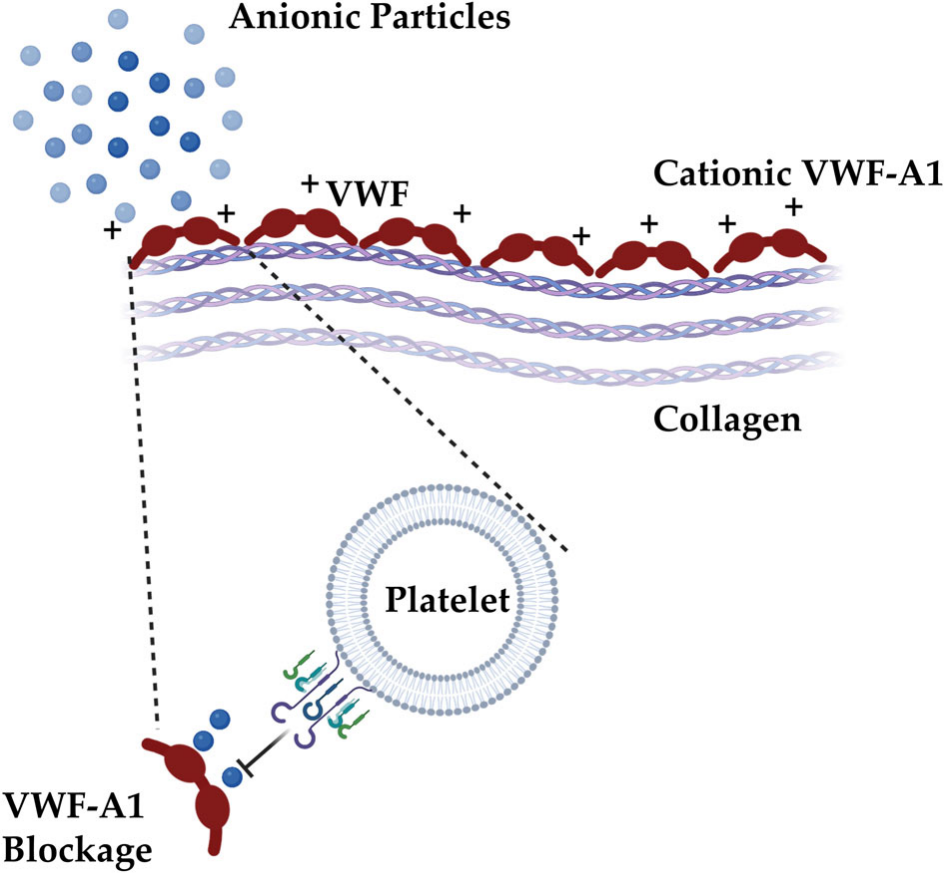
Particles can interfere with platelets' aggregation via physio‐chemical inhibition of platelet VWF interactions: Negatively charged particles can interact with positively charged VWF‐A1 and block its availability for platelet interaction. Created with BioRender.com.

Interestingly, in a more recent study by Griffin et al.^19^ the authors showed that 50–60 mV negatively charged NPs of different matrices exhibited an antithrombotic response in an in vitro microfluidics‐based thrombosis assay. The concept behind using these specific NP size and charge relies in the arterial platelet thrombosis cascade, where Von Willebrand factor (VWF) seems to play a key role. During the early stages of arterial thrombosis, VWF elongates under critical high shear stress under flow and unveils its interacting domains. Following VWF elongation, VWF A1 domain interacts with platelet's GP1b receptor to initiate the cascade. To counteract the exposure of positively charged A1 domains upon elongation of VWF by pathological shear stress, the researchers perfused negatively charged small NPs^19^ on type I fibrillar collagen‐coated microfluidics chips (100 μg/ml). Their results showed that negatively charged PS NPs can inhibit the thrombotic occlusion time in a microfluidic assay operating under arterial high shear conditions in the presence of collagen, mimicking thrombosis at sites of atherosclerotic plaque rupture. The authors proposed that the antithrombotic effect of these particles stem from their potential to physically alter the tertiary structure of VWF^19^ (see Figure 3).

Particle size is also one of the critical parameters to be investigated. One of the early studies in this field was conducted by Miyamoto et al.^20^ The study examined PS particles of sizes ranging from 0.2 to 1.1 μm with all examined PS particle sizes inducing platelet aggregation and ATP release when they were incubated with washed platelets in HEPES‐buffered Tyrode's solution. Moreover, the study also emphasizes the crucial effect of the ratio between platelets and particles that affects platelet aggregation. Furthermore, smaller particles were engulfed by platelets, while larger particles were enveloped by platelet pseudopodia.^20^

In another study, Mayer et al.^21^ several PS NPs of varying sizes (20–220 nm) and modifications were used to investigate the role of NP size in hemocompatibility. Two factor levels were measured, that is, β‐thromboglobulin (β‐TG) and Platelet Factor 4 (PF‐4) levels, following the introduction of the NPs. Amide PS NPs of 220 nm and 14.7 mV zeta potential show the highest levels of factor 4, followed by carboxylated NPs of 26 nm with a zeta potential of −44.9 mV and by carboxylated NPs of 160 nm and a zeta potential of −47.6 mV. The main conclusion of this study was that activation of platelets by PS NPs occurs when a sufficient high concentration of particles is mixed with thrombocytes, independently of the net surface charge.

It is worth noting that the interaction between platelets and particles may be also influenced by protein corona adsorption on the particle's surface. A unique study by Lundqvist et al.^22^ showed that different PS NPs of size and charge, have different protein corona map on their surface. The study examined 50 and 100 nm PS NPs of different charge nature and showed that even for a fixed material matrix, the effect of the size and charge is crucial in determining the protein array found in the corona. The latter may have a major effect on hemocompatibility and on platelet tolerance.

As a final remark to this section, we note that although PS NPs have been widely studied with valuable insights, such particles nevertheless come short of being suitable for in vivo application due to their limited biodegradation and biocompatibility.

### 2.2 PLGA NPs

Poly(lactic‐co‐glycolic) acid (PLGA) has been gaining significant attention in the medical field, as it offers ample characteristics that can be tailored for various medical application and for controlled drug delivery. PLGA is biocompatible and biodegradable polymer, and its surface can be chemically modified to allow the coupling of various ligands.^23^ PLGA microparticles and NPs are widely recognized as effective vehicles for targeted drug therapy and controlled drug release.^24^, ^25^ Furthermore, PLGA polymer can be modified to prolong its circulating half‐time in blood. While the biocompatibility of the PLGA is well‐established, the behavior of the PLGA NPs with blood components, particularly platelets, remains relatively unexplored.^26^ To examine platelets‐particles interactions, PLGA, PLGA‐macrogol and chitosan, within the size range of 500 nm to 10 μm were fabricated and tested by Ramtoola et al.^26^ In their study, a series of ascending concentrations of the particles' suspension (0.01–500 mg/ml) were mixed with plasma rich platelets (PRP) and then assessed by light‐transmission platelet aggregometry. The concentration range was chosen to cover the potential particle concentration that may raise safety concerns. Their study shows that PLGA particles did not induce neither aggregation nor inhibition of platelet function which indicate their safety and biocompatibility.^26^

Following this research, two successive studies were conducted by Bakhaidar et al.^3^, ^27^ to investigate the effect of size‐selected PLGA‐PEG (PEG—polyethylene glycol) NPs on washed platelets' activation and aggregation. The findings of these studies were inconclusive as the smallest NPs (i.e., 112 nm), exhibited a tendency to activate platelets while larger NPs (i.e., 348 and 576 nm), at the highest concentration, exhibited a tendency to delay platelet aggregation. Notwithstanding, all tested PLGA‐PEG NPs did not interfere with platelet activation status nor inhibited their aggregation in response to thrombin.^2^

These findings align with prior research by Li et al.^28^ which reported that PLGA, chitosan–PLGA and a series of chitosan NPs did not modify platelet aggregation at a concentration below 10 μg/ml. However, at a high concentration of 100 mg/ml, PLGA and chitosan particles weakly down‐regulated platelet aggregation, which may indicate an anti‐thrombotic effect.

Another study by Griffin et al.^19^ employed negatively charged 115 nm in size PLGA NPs in a high shear microfluidic model. The models were coated with VWF and perfused with whole blood and negatively charged particles. They showed that treated blood reduced thrombus formation by 10‐fold, most likely as a result of the particles' interacted with the VWF, causing interference with VWF‐platelet interaction. This was also shown to reduce thrombus stability without increasing bleeding in mice.^19^

### 2.3 Carbon NPs

Carbon nanomaterials are a family of particles known for their physiochemical characteristics and for being used for bioimaging in the body.^29^ As the medical applications of engineered carbon NPs continue to increase, it becomes essential to investigate their pharmacological effects.^30^ Furthermore, with the growing production of carbon NPs, there is a major concern about their systemic intake through inhalation. Nemmar et al.^31^ showed that there is a very rapid systemic translocation of nano‐sized particles from the lung to peripheral blood and therefore, toxicity and biocompatibility of such particles need to be delineated.

**FIGURE 4:**
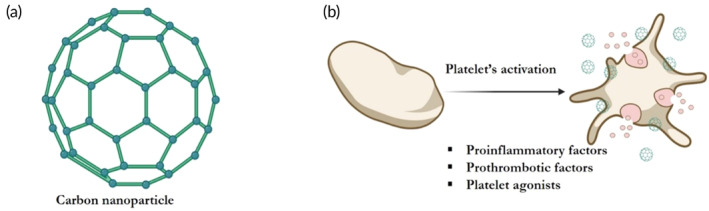
Carbon nanoparticles interactions with platelets. (a) Schematic construct of a carbon nanoparticle. (b) Carbon nanoparticles can interact with platelets' membrane and cause platelet degranulation and secretion of certain factors (see main test for details). Created with BioRender.com.

Radomski et al.^32^ examined different carbon NPs derivatives both in vitro and in vivo. The authors mixed the following carbon NPs with washed platelets to examine their activation potential: multiplewall (MWNT), singlewall (SWNT) nanotubes, C60 fullerenes (C60CS) and mixed carbon NPs (MCN). Their findings suggest that MCN, MWNT, and SWNT induce platelet aggregation in a concentration‐dependent manner, while C60CS does not. Interestingly, the different carbon NPs induced platelet aggregation via different cascades, such as ATP release, degranulation, over expression of α_II_bβ_3_ receptor and protein kinase C (PKC) aggregation‐dependent pathway^32^ (see Figure 4 and Table 1 for details in Radomski et al.^32^). For example, MCN induced ATP release and degranulation while MWNT did not. To verify these findings in vivo, a FeCl_3_ induced carotid artery thrombosis was created in rats and development of vascular thrombosis was monitored following exposure to different carbon NPs. The results demonstrate that MCN, SWNT and MWNT NPs significantly accelerated the time and rate of development of carotid artery thrombosis, while C60CS exerted no significant effect on vascular thrombosis development.^32^ In conclusion, each carbon NP formulation should be evaluated individually to carefully assess their potential side effects and ensure public safety.

### 2.4 Silica NPs

Silica NPs, or amorphous silica, are an attractive candidate for different applications in various medical fields due to their high thermal and chemical stability and biocompatibility.^33^ Bharali et al.^34^ and Roy et al.^35^ developed organically modified silica (ORMOSIL) NPs as a nonviral vector for efficient in vivo gene delivery and cancer photodynamic therapy. Due to the increasing interest of silica NPs, there is a need to define the cytotoxicity profile of these particles upon administration to the human body.

**FIGURE 5:**
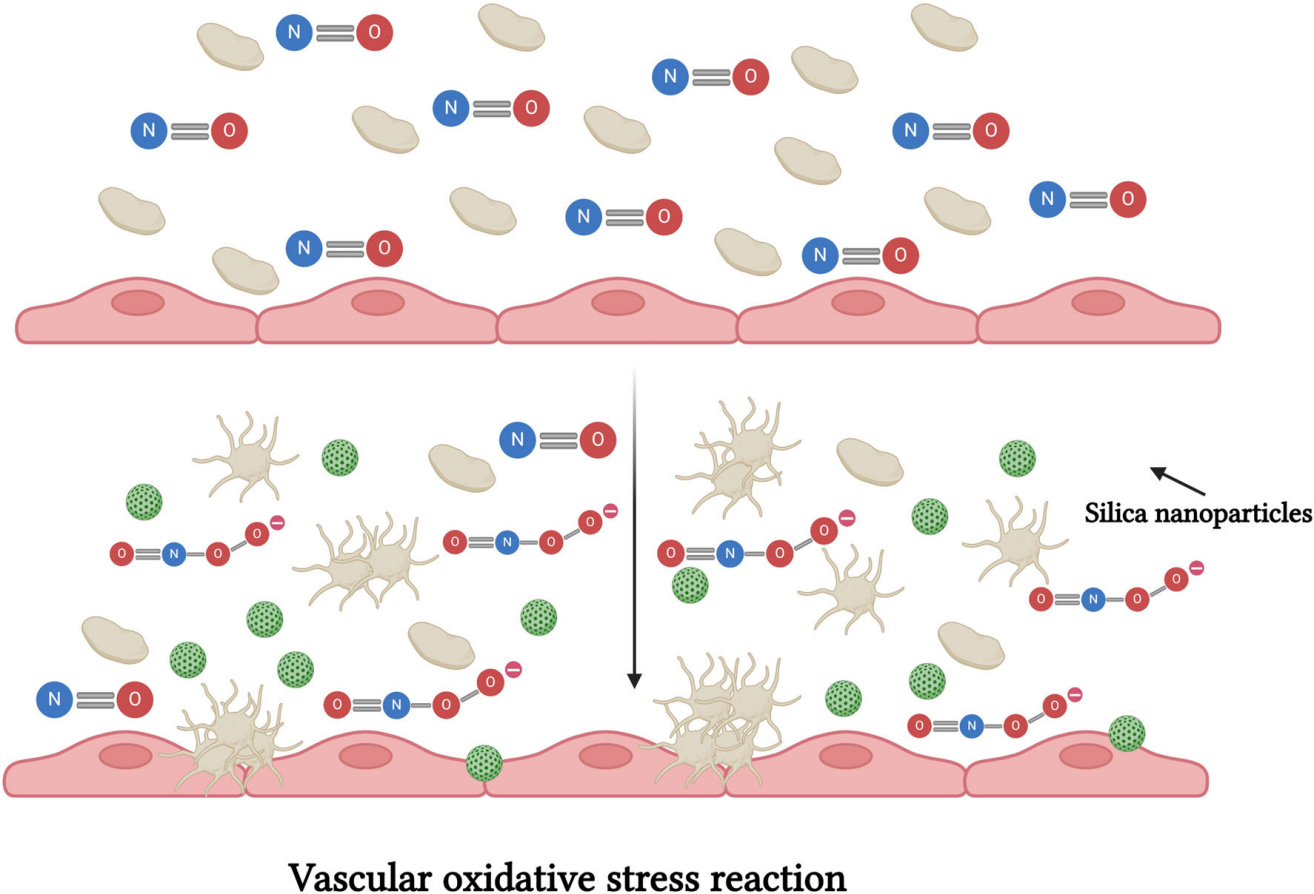
Silica particles can interfere with platelets via the vascular oxidative stress reaction. Silica particles interfere with nitric oxide bioavailability in a reaction called vascular oxidative stress reaction and lower its concentration. Lowering nitric oxide concentration increases the risks to atherosclerosis. Created with BioRender.com.

Duan et al.^36^ proved that Silica NPs (SiNP) have a devastating effect on vascular endothelial cells. In their latest study from 2018, the authors studied the effect of 63 nm SiNPs on zebrafish embryos and found that these particles disturbed the cytoskeleton organization, increased proinflammatory and procoagulant factors. Moreover, SiNPs decrease blood flow thereby facilitating blood aggregates in the caudal vein. Silica NPs were found to impact vascular homeostasis through their interaction with platelets, specifically affecting the bioavailability of vascular nitric oxide (NO), as illustrated in Figure 5. NO is a potent vasodilator and vasorelaxant that also exerts antiplatelet effects. Any impairment in NO or decrease in its concentration, disrupts vascular homeostasis and can lead to platelet aggregation.^37^

The superoxide anion is an oxygen radical anion that reacts with nitric oxide to produce peroxynitrite, ONOO^−^. The later reaction is known as vascular oxidative stress reaction that decreases the bioavailability of NO. Evidence emphasizes the major role of endothelial NO concentration in atherosclerosis pathogenesis, therefore lowering its bioavailable concentration increases the risk of vascular diseases.^38^

Corbalan et al.^39^ explored the noxious effects of amorphous silica (10–40 nm) NPs on human endothelial cells. They showed that amorphous silica NPs trigger nitric oxide/peroxynitrite imbalance leading to endothelium inflammation and necrosis. Inspired by the noxious effect of amorphous silica on the endothelium, research aimed at investigating the hemocompatibility of amorphous silica NPs continues. Silica NPs with sizes of 10, 50, 150, and 500 nm were incubated with human platelets where it was found that silica NPs lower the ratio of [NO]/[ONOO–] by stimulating ONOO– production. Furthermore, silica NPs upregulate the expression of P selectin and α_II_bβ_3_, both of which indicate platelet activation. The results also support a positive relationship between NPs concentration and platelet activation. Notwithstanding, the effect on platelets were inversely proportional to NP size (Table 2).

**TABLE 2:** Methods and mechanism involved in platelet‐NPs interaction of different NPs types.

Type of NPs	Methods	Methods and main results	Possible mechanism involved
Polystyrene latex NPs	In vitro In vivo Microfluidics	In vivo—studies in hamsters, negatively charged PS NPs reduced thrombus formation, while positively charged increased its formation.^13^ In vitro—both positively and negatively charged NPs interacted with platelets and increased aggregation in PRP.^18^ In microfluidics—In high shear stress thrombus microfluidic model negatively charged NPs had antithrombotic effect with whole blood19.	Electrostatic interactions between platelet's surface and the amine groups on the particle, see Figure 2c. Negatively charged NPs may induce ADP platelet aggregation in vitro. Another mechanism suggested in high shear models, is that negatively charged NPs interact with VWF, as seen in Figure 3, therefore reducing thrombus formation.
PLGA NPs	In vitro Microfluidics	In vitro—High concentration of modified PLGA down regulated platelet aggregation in vitro.^3^ Microfluidic—Negatively charged PLGA NPs were perfused through high shear thrombus microfluidic model, decreased thrombus development.^19^	These phenomena may be the result of physical interference of the NPs, thus preventing from platelets to interact with each other.^3^ The suspected mechanism for reducing thrombus formation is attribute to the interaction of the negatively charged particles to VWF at high shear rate.^19^
Carbon NPs	In vitro In vivo	In vivo & in vitro‐ MCN, SWNT, and MWNT induced platelet aggregation in vitro and thrombus formation in rats, while C60CS did not.^32^	Carbon NPs induce platelet aggregation via different pathways such as: ATP release, degranulation, over expression of αIIbβ3 receptor and protein kinase C (PKC) aggregation‐dependent pathway. For example, MCN induce ATP release and degranulation,^32^ as seen in Figure 4.
Silica NPs	In vivo In vitro Microfluidics	In vivo—SiNPs caused hemodynamic changes and aggregation in the caudal vein of zebrafish.^36^ In vitro—SiNPs decreased NO levels and increased P‐selectin levels in human ECs model leading to platelet aggregarion.^37^ Microfluidics—Preincubation of EC with SiNPs increased platelet adhesion under flow condition in microfluidic model.^41^	SiNP reduced blood flow in zebrafish thus increasing blood coagulation.^36^ In in vitro model SiNPs activated platelet via increased expression of P selectin and αIIbβ3, Figure 5.^37^ In an endothelialized microfluidic model SiNP increase PECAM expression which supports platelet adhesion.^41^
PAMAM dendrimers NPs	In vivo In vitro	In vivo—Positively charged dendrimers had deadly effect on mice.^44^ In vitro—Dobrovolskia showed that dendrimers of G5‐G6 with positive charge induced platelet aggregation.^43^	The fatal outcome may be a result of low levels of fibrinogen and high levels of fibrin degradation products.^44^ Large and positively charged dendrimers induce platelet aggregation by disturbing the membrane integrity.^43^
Gold NPs	In vitro	5–30 nm gold NPs did not induce platelet aggregation, however, 60 nm NPs inhibited platelet aggregation.^54^	60 nm particles have dose‐dependent inhibition effect of ADP‐induced platelet aggregation.^54^
Silver NPs	In vivo In vitro	In vivo—Preincubation of platelets with silver NPs reduced their adhesion to thrombogenic surfaces in mice.^51^ In vitro—There are contradicting results regarding silver NPs. While some studies have shown that silver NPs have antiplatelet characteristics^60^ others found them to increase thrombus formation.^58^	Elevated calcium levels and expose P selectin. Antiplatelet characteristic may be attributed to inhibition of integrin‐mediated platelet aggregation.^51^
Iron Oxide NPs	In vitro	Negatively charged particles were incubated with platelets at different concentration and induced dose dependent aggregation.^63^	ADP dependent platelet activation.^63^
Zinc Oxide NPs	In vivo In vitro	In vivo‐ Zinc NPs reduced bioavailability of coagulation factors in rat's blood.^69^ In vitro—Negatively charged particles increased platelet aggregation when incubated with PRP.^71^	Zinc oxide particles adsorb coagulation factors.^69^ Delay thrombin production. Membrane interaction between NPs and erythrocyte and platelets.^71^
Nickel oxide NPs	In vitro	62 nm NPs caused morphological changes to platelet in vitro.^60^	Promote fibrin polymerization.^60^
Copper NP	In vitro	Negatively charged NPs increased aggregation formation.^63^	Interaction with P2Y_12_ receptor.^63^
Titanium dioxide NPs	In vitro	TiO_2_ anatase induced platelet aggregation in vitro, and TiO_2_ rutile did not.^71^, ^77^	Unclear
Platinum NPs	In vitro	Two types of platinum NPs were incubated with washed platelets and in PRP. Rapid aggregation was formed with washed platelets; however, no aggregation was found in PRP.^3^	Passive platelet interaction and active via αIIbβ3 receptor.^3^

Silica NPs effect on the coagulation cascade is not merely the results of stimulating the vascular oxidative stress reactions but also related to blood coagulation factors and plasma proteins such as fibrinogen and fibronectin. Modulating their bioavailable concentration and function by adsorption can impact the thrombosis cascade.^40^

The effect of SiNPs on platelets was also studied in an endothelialized microfluidic model. Endothelial cells were preincubated with 250 nm particles, causing changes in the protein expression of endothelial cells and overexpression of PECAM, resulting in increased platelet adhesion.^41^

### 2.5 PAMAM dendrimers

Dendrimers are regularly branched polymeric macromolecules that are radially arranged from core to periphery, resembling a tree.^42^ Dendrimers are produced by gradually assembling dendritic polymers around a central core. The dendrimer's generation (*G*) is determined by the total number of branching steps added by each reaction during the synthesis process. There are two categories for dendrimers: low‐generation (*G* < 4) and high‐generation (*G* ≥ 4). Generation zero, or *G*0, is a term occasionally used to describe the dendrimer's central core.^43^

Dendrimers are gaining wide recognition and increasing research interest due to their versatile characteristics applicable in biomedical applications. The multivalency of dendrimers is one of their most exploited features, offering various applications. As dendrimers radially grow, their molecular weight, generation, and branches also increase. This can offer many opportunities for drug loading and vectoring as drugs can be encapsulated inside the dendrimer core or immobilized on its branches.^42^

In the 1980's, Tomalia et al.^43^ reported that Polyamidoamine (PAMAM) dendrimers can be completely synthesized and commercialized. The size of PAMAM dendrimers can range from 1 to 14 nm (from G0 to G10). Later, in the 1990s, studies revealed that PAMAM dendrimers possess a hollow core that can be used to encapsulate small molecular weight hydrophobic drugs. PAMAM dendrimers also exhibit antimicrobial properties with potential applications in various medical fields.

Due to their versatile applications, PAMAM dendrimers' hemocompatibility has been widely investigated. Dobrovolskaia et al.^45^ tested the effect 12 different formulations of PAMAM dendrimers on human platelets suspension in vitro by adding and incubating these NPs with plasma rich platelets (PRP) and performing light transmission aggregometry. The dendrimers are of different generations and group termination thus vary in size and surface charge. The study shows that large positively charged dendrimers of G5‐G6 induce platelets aggregation. However, small G3 cationic PAMAM dendrimers, anionic and neutral dendrimers of all sizes, did not induce platelet aggregation. The study also demonstrates that large cationic dendrimers induce platelets aggregation through disrupting the integrity of cell membrane. It was also interesting to reveal that G3 cationic dendrimers increase the expression of CD62P receptor on platelets' surface thus, indicating an induction of platelets' activation status.^45^

In another in vivo study conducted by Greish et al.^46^ several dendrimers of different generations and surface charges were examined. The authors fabricated hydroxyl (OH), amine and carboxyl‐terminated dendrimers of several generations (G3.5–G7). These dendrimers were intravenously administered to mice while their effect on blood was monitored. The results of this study are consistent with the findings that we have previously mentioned; the cationic dendrimers were fatal at doses >10 mg/kg causing hematological complications such as coagulation whereas negatively charged and neutral dendrimers of similar sizes were tolerated at 50‐fold higher doses.^46^

**FIGURE 6:**
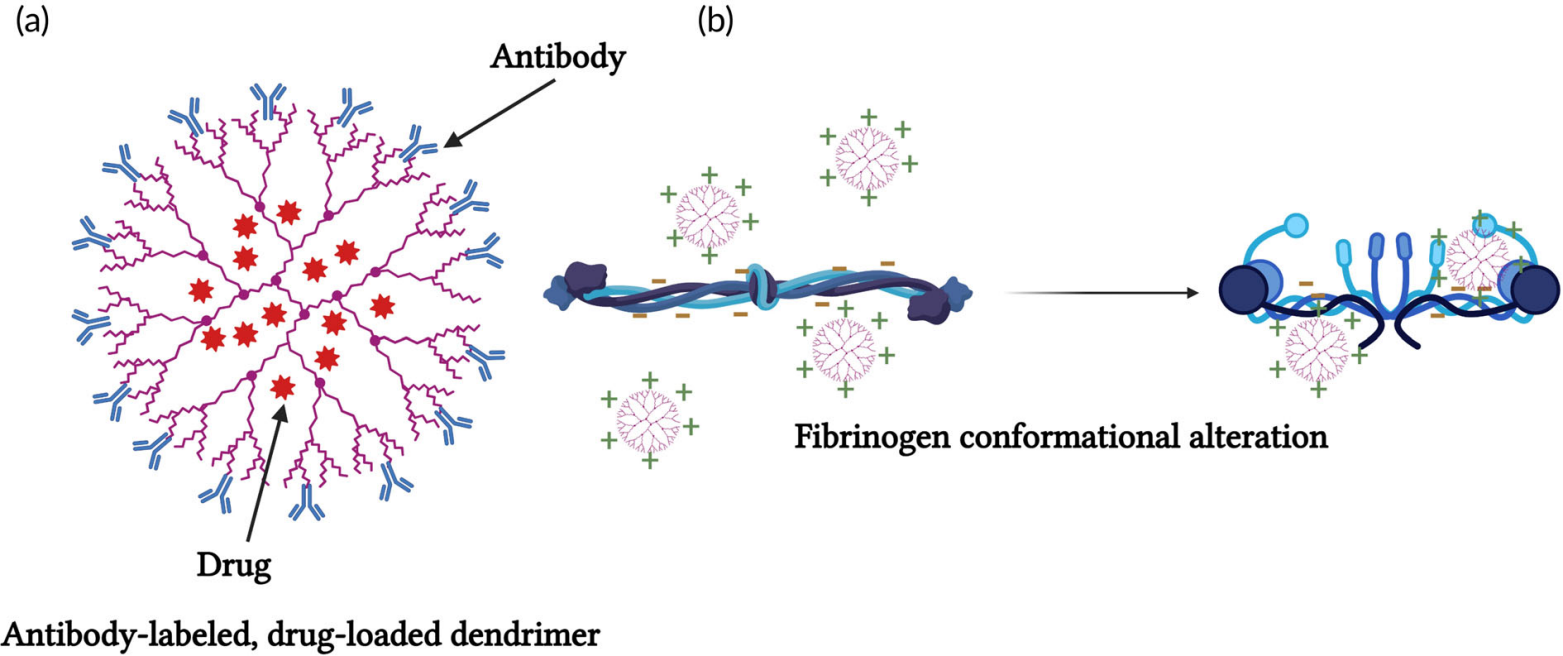
Dendrimers interactions with platelets. (a) Schematic representation or dendrimers, exhibiting branched polymer macromolecules with versatile applications. For example, drugs can be encapsulated inside the dendrimer while recognition tags such as antibodies can be linked to its surface. (b) Positively charged dendrimers can interact with negatively charged fibrinogen via electrostatic interactions and alter its conformational state. These interactions escalate fibrinogen aggregates in a thrombin‐independent manner resulting in a formation of dense and high molecular weight fibrinogen aggregates. Created with BioRender.com.

These findings complement a later study by Jones et al.^47^ in which the effect of cationic, anionic and neutral dendrimers on platelets was studied. Shortly summarizing, the study demonstrated that cationic G7 PAMAM dendrimers activate platelets and dramatically alter their morphology. These changes to platelet morphology and activation state substantially altered platelet function, including increased aggregation and adherence to surfaces. The same group later on, further analyzed the mechanism by which the PAMAM cationic dendrimers induce platelet activation.^47^ Their findings suggested that electrostatic interactions occur between the dense cationic charged dendrimer and the negatively charged fibrinogen. These interactions escalate fibrinogen aggregates in a thrombin‐independent manner resulting in a formation of dense and high molecular weight fibrinogen aggregates,^47^ see Figure 6; such findings are also consistent with Watala et al.^48^

While some studies focused on high generation PAMAM dendrimers, a recent study focused on low generation anionic and cationic PAMAM dendrimers.^49^ The main results of this intensive study complement previous studies. Cationic dendrimers increased prothrombin time and changed the conformation and coagulation of fibrinogen while anionic ones did not induce any of these effects. Moreover, the effects of the dendrimers were enhanced with increasing generation.^49^

To summarize so far, dendrimers' effect on platelets can be relatively predicted in a simpler manner than that of NPs'.

## 3 METALLIC NPs

The fields of biomedical sciences and engineering have witnessed a revolution in the development of metallic particles over the years. Since their initial discovery, metallic particles have been extensively investigated to address unresolved health‐related issues that conventional medicine cannot tackle. Metallic particles possess brilliant potential in nanotechnology owing to their versatile physical and chemical characteristics. Nowadays, metallic particles can be easily synthesized and modified with various biological moieties for theranostic purposes, such as magnetic affinity chromatography, Magnetic Resonance Imaging (MRI), optical imaging, targeted drug delivery, and gene delivery.^50^, ^51^ As the demand for applications increases, there is also an increase in deciphering their hemocompatibility, especially platelets. In this section, we are reviewing most common metallic particles in medicine, highlighting their clinical applications, and investigating their effect on platelets.

### 3.1 Gold NPs

Gold NPs are among the most investigated NPs due to their rich physicochemical properties that endow them with a broad spectrum of potential biomedical application specially in imaging and targeted thermal therapy.^52^ Due to their importance in medical applications, platelets' compatibility was intensively investigated.

Throughout the literature, the effect of gold NPs varies distinctively as a function of concentration, surface charge, shape and size. The gold NPs properties also affect their proteins' corona which alters the interactions with platelets. One of the early studies in this field by Dobrovolskaia et al.^53^ examined two colloidal gold NPs of 30 and 50 nm. The study tested the alteration of particle's hydrodynamic size and charge following plasma incubation, proteins' corona map on particle's surface and collagen‐induced platelet aggregation. The study reported several findings. First, the hydrodynamic size of colloidal NPs increases following plasma incubation. The latter is attributed to plasma proteins adsorption to particle's surface due to electrostatic interaction, inter alia. Second, the measured zeta potential of these particles was highly negative before plasma incubation, however, the charge was approximately neutralized following protein adsorption. Third, the detected protein corona map on the 30 nm particles and on the 50 nm particles were different. Mass spectrometry showed that the 30 nm colloids bind a greater spectrum of proteins which may be attributed to surface area differences. Lastly and most importantly, both colloids inhibited collagen‐induced platelet aggregation at a concentration of 0.45 and 0.42 mg/ml for the 30 and 50 nm, respectively. Another study that showed similar effect of gold NPs discussed the effect of size of gold nanospheres coated with components of autologous blood plasma on ADP‐induced platelet aggregation.^54^ The study examined 5, 10, 20, 30, and 60 nm gold NPs of both versions: coated and non‐coated. The study suggested that the gold NPs of 5–30 nm size have no effect of ADP‐induced platelet aggregation in the concentration range examined (i.e., 1.6, 2.0, and 5.0 μM), regardless of surface coating. However, the 60 nm particles showed dose‐dependent inhibition effect of ADP‐induced platelet aggregation. It is worth noting that the findings of this study are not consistent with other studies in this field; the discrepancy in results may be attributed to the NPs preparation method that affects the particles' physicochemical properties.

Other studies show the opposite effect of pro‐aggregation potential. One of these studies, conducted by Ajdari et al.^52^ in which they examined colloidal gold NPs in the range 12 to 85 nm demonstrated that the effect of such particles is a function of concentration and surface chemistry. Namely, 1.2 mM colloidal gold NPs have no effect in the blood while increasing the concentration to 5 mM show pro‐thrombotic dependent effect. Moreover, arginine‐glycine‐aspartic acid (RGD) modified particles show pro‐coagulant effect while PEG‐thiol, human fibrinogen and clopidogrel modified gold NPs, prevented blood clot formation.^52^

So far, we mentioned several studies that examined the effect of different gold NPs, however, we did not mention the effect of the chemical stabilizers, which are used in NP synthesis, on platelet aggregation. Stabilizer agents are used in bottom‐up methods to fabricate NPs. The main aim of a stabilizer is to control the growth of the NPs and impede agglomeration.^55^

Such a study was conducted by Hecold et al.^55^ in which they evaluated the effect of two chemical stabilizers on platelets activation status. The study dealt with two chemical stabilizers, polyethylenimine (PEI) and polyvinylpyrrolidone (PVP). The authors fabricated two sizes of NPs using two different stabilizers. The diameter of gold NPs stabilized by PEI was approximately 20 nm, while the diameter of gold NPs stabilized by PVP was 50 nm. The particles, in different concentrations, were incubated with whole blood to check their effect on platelet's activation status. Both the morphology and growth factors secreted from platelets were analyzed. The results show that both NPs activate platelets which is manifested in morphology changes of filopodia and lamellipoda and higher concentration levels of growth factors.^55^ Even though the exact mechanism behind this phenomenon is not known yet, these are important findings for future medical applications.

We conclude this section by underlining that to date there are still no studies demonstrating nor predicting the effect of gold particles considering all the potential variables that can affect platelets activation and aggregation. However, particle hydrodynamic diameter seems to play a crucial role therefore, each case should be investigated individually to assess the potential effects.

### 3.2 Silver NPs

Silver NPs have gained a widespread reputation due to their antimicrobial properties. This noble metal has unique chemical and physical properties most attractive in developing biomedical applications.^56^ In parallel, silver NPs have numerous anti‐microbial applications in distinctive fields such as cosmetics and food storage.^56^ The growing demand for silver NPs raises many skeptics regarding their toxicity and blood compatibility; in turn, numerous studies have focused on investigating these crucial aspects. As mentioned earlier, the effect of NPs on blood cells and body tissues remains controversial as each NP possesses a unique matrix of physicochemical properties that can differently affect blood compatibility. In this context, silver NPs are of no exception as several studies have reported conflicting outcomes.^56^ Shrivastava et al.^57^ have synthesized spherical shape 10–15 nm silver NPs. The authors demonstrate the anti‐platelet potential of these NPs at a concentration of 50 μM. Silver NPs inhibited integrin‐mediated platelet aggregation, secretion and adhesion to collagen or fibrinogen coated surfaces. Moreover, silver NPs retracted fibrin clot in a dose‐dependent retraction manner regardless of agonists used.^57^ It is also important to mention that silver NPs suppressed prothrombotic cascades that could have resulted from activated platelets. In this context, pre‐activated platelets, obtained from patients, were perfused on collagen‐coated surfaces. The study showed that activated platelets, which were incubated with silver NPs, adhered less on thrombogenic surfaces that physiologically recapitulate in vivo conditions.^57^ Paradoxically, Jun et al.^58^ reported that silver NPs enhance thrombus formation and platelet procoagulant activation. In their study, the authors used silver NPs of <100 nm diameter. The particles induced platelet aggregation in human washed platelets upon incubation with silver NPs, in a concentration‐dependent manner.

The platelet procoagulant activity, which is an important contributor to clot formation, was also measured. This marker was evaluated by measuring phosphatidylserine expression level on platelet's surface. The results indicate an increase in phosphatidylserine levels in a concentration‐related manner.^58^ Furthermore, other key markers for aggregation and coagulation, for example calcium levels were measured. Results show that platelet treatment with silver NPs increases intracellular calcium levels. Other markers such as P‐selectin exposure and serotonin release were also measured and an increase in their level was recorded following incubation with silver NPs. Lastly, it is crucial to indicate that the in vivo result in this study is consistent with its in vitro as exposure to silver NPs enhanced venous thrombus formation following particles injection into rat via intra venous administration.^58^ Other studies show similar effects of silver NPs upon contact with platelets. Krajewski et al.^59^ examined silver NPs of 10–15 nm and showed concentration‐related activation of platelets at a concentration of 30 mg/L. following particles contact with platelets, both β‐thromboglobulin and P‐selection levels were significantly higher compared to control group. Another paper, Guildford et al.^60^ examined the effect of 90–240 nm silver NPs on platelet activation and documented changes in platelet morphology using scanning electron microscopy (SEM).

Contrary to previously stated studies, other studies document no effect on platelet activation and pro‐coagulation upon exposure to silver NPs. Such findings are documented in Huang et al.^61^ and Smock et al.^62^ The latter study examined the effect of 20 nm silver NPs coated with either polyvinyl pyrrolidone (PVP) or citrate. The authors state that neither PVP nor citrate‐coated silver NPs show effects on platelet aggregation, coagulation process, or activation at a concentration up to ~40 μg/ml. Smock et al.^62^ evaluated platelet aggregation following a daily ingestion of silver oral colloidal silver NPs for 2 weeks. Platelets were collected, and their aggregation potential was evaluated using light transmission aggregometry where collagen and ADP were used as agonists. The findings demonstrate that platelet activation was not detectable at particle concentration of <10 μg/L.

### 3.3 Iron oxide NPs

Iron oxide, Fe_2_O_3_, is one of the highly acclaimed and well‐studied metals for biomedical applications.^50^ Several unique characteristics endow iron oxide with its wide reputation. Its most exceptional trait is the naturally paramagnetic feature that is extensively used in enhanced resolution contrast agents for magnetic resonance imaging (MRI).^50^ Moreover, iron oxide particles are also used in targeted drug therapy, cancer targeting by hyperthermia, magnetic separation for affinity chromatography, molecular and cellular tracing, and detection of inflammation due to several pathological conditions.^50^

Due to their ample medical applications, many surface coating technologies were developed to increase iron oxide particles hemocompatibility. Such coatings are PEG and dextran. Beside hemocompatibility, it is crucial to investigate the effect of these particles on platelets as any mal regulation in their function can lead to deadly outcomes, such as stroke and myocardial infraction. Extensive studies in this field were conducted and the results are not unequivocal as multiple parameters can contribute differently. Here we present some of these studies and highlight their conclusions.

Deb et al.^63^ fabricated iron oxide NPs with mean hydration diameter of 11 nm and negative zeta potential of −6.58 mV. Iron oxide NPs, of different concentrations, were incubated with plasma rich platelets (PRP) and then exposed to ADP. The results show that iron oxide NPs possess pro‐aggregation potential that is strongly affected by their concentration; higher NP concentrations induce higher aggregation. Moreover, results also show that platelet aggregation, induced by iron oxide NPs, is mediated by ADP receptors on platelet's surface.^63^

In another study by Guildford et al.^63^ two types of iron oxide NPs were fabricated: Fe_3_O_4_ and Fe_2_O_3_, which are materials that are used in stents and implants, were fabricated with sizes of 20–30 nm and 55–65 nm size, respectively. Both NPs induce platelet aggregation in an in vitro assay, using SEM.^60^

In 2012, Aurich et al.,^64^ developed a novel method to magnetically label platelets with iron oxide particles. This new method can substitute radioactive labeling of cells that is no longer accepted in many countries worldwide. The study used contrast agent for MRI that is FDA approved, for example, ferucarbotran. Ferucarbotran particles of 92 nm diameter and −36.9 mV zeta potential was examined. The effect of ferucarbotran on platelets was concentration‐dependent effect. At a high concentration of 10 mM, the particles impair platelet function however, at lower concentration of 0.5 mM, the particles had a minor impact on platelet activation status.^65^

In contrast, Bircher et al.^66^ synthesized carbon‐coated iron oxide NPs of 30 nm and then functionalized them all using PEGylation agents with different end groups such as OCH_3_, NH_2_, COOH, human IgG and ProteinA‐protected IgG. Different concentrations of particles were mixed with blood and clot formation time was measured. The study demonstrated that all PEGylated nanomagnets have no observable influence on coagulation parameters at a concentration of 0.5 mg/ml, as opposed to SiO_2_ particles which served as positive control.

### 3.4 Zinc oxide NPs

Zinc oxide is the second most abundant metal oxide following iron oxide.^67^ Extensive studies were conducted to evaluate its potential application in numerous fields. The physicochemical properties of zinc oxide make it an excellent candidate for biomedical applications, especially being labeled as FDA‐approved metal oxide. These applications include chemotherapy agents, drug and gene delivery, vaccine adjuvants, and biosensors.^68^, ^69^ Therefore, the hemocompatibility of zinc oxide should be evaluated. Size and surface properties of a NP are among the most crucial properties that dictate its hematological potential. Yang et al.^69^ fabricated 20 and 100 nm zinc oxide NPs with three surface modifications (pristine, L‐serine, and citrate). Due to different surface coating, the zeta potential also varies between particles as citrate is negatively charged and L‐serine moieties are positively charged. To assess their hemocompatibility, NP particles were administered into Wistar rat blood vessels while coagulation time, thrombin generation assay and measurement of the levels of coagulation factors were all evaluated. The study indicated that zinc oxide NPs can adsorb blood coagulation factors and lower their bioavailable concentration in the blood stream. The coagulation factors adsorbed to particle's size can be a function of particle's charge and size, however, all these particles show common adsorption profile. As a result of this, a delay in thrombin generation times and potentials were observed.^69^ Another study, Haberl et al.^70^ examined different metal‐based NPs and zinc oxide particles among them. The particles were injected into the murine microcirculation and were not found to affect thrombus formation. On the other hand, zinc oxide of negatively charged 1820 and 430 nm particles show pro‐aggregatory effects by activating human platelets following 3 h incubation in plasma rich platelets.^71^

### 3.5 Nickel oxide NPs

Nickel oxide has limited biomedical applications as most of them are classified in energy industry domain such as battery electrodes, gas sensors, and fuel cells.^72^ Only limited papers examined their hemocompatibility and found that nickel oxide NPs of 62 nm induce morphological changes in platelet shape.^60^

### 3.6 Copper NPs

Copper NPs have also limited biomedical applications due to their non‐stability and over sensitivity to oxygen and water.^73^ Therefore, many strategies have been developed to increase their stability and decrease their reactivity upon exposure to water and oxygen. Copper NPs may be used as antimicrobial and antibacterial agents however, they are less prominent compared to silver NPs in this context.^74^ Due to their restricted uses, little is known about copper NPs hemocompatibility, especially with platelets. one of few studies that investigated copper NPs was Deb et al.^63^ The authors fabricated negatively charged copper NPs of 10 nm and investigated their effect on platelets aggregation. The findings of this study demonstrate that these have pro‐aggregatory effects by targeting P2Y_12_ receptor.

### 3.7 Titanium dioxide and platinum NPs

Titanium dioxide is an inorganic compound that gains its wide reputation due to its photoactivity.^75^ Therefore, titanium dioxide is widely used in sunscreen industry.^76^ The increased demand for titanium NPs development in medicine fields stems from the intensive advances in photodynamic therapy such as photosensitizers.^75^ Motivated by the future applications and substantial potential for human exposure, several studies were conducted to evaluate titanium dioxide health‐ related issues. Titanium dioxide naturally occurs in several compositions such as anatase and rutile and their physicochemical features vary accordingly. Haberl et al.^70^ examined TiO_2_ anatase and TiO_2_ rutile NPs of 423 and 309 nm, respectively. They showed that TiO_2_ anatase significantly enhanced platelet aggregation in mice blood while TiO_2_ rutile NPs did not. Consistent with this study, Šimundić et al.^71^ showed that TiO_2_ anatase activate platelets.

Platinum NPs have brought increased attention in medical fields as many applications could benefit from their intrinsic catalytic properties.^77^ For example, Zia et al.^2^ fabricated two negatively charged platinum NPs of 7 and 73 nm. The particles were incubated with washed platelets in Tyrode's‐HEPES buffer and in PRP. When both particles incubated with human washed platelets, rapid aggregation occurred. However, when particles were incubated in PRP, to closely recapitulate physiological conditions, both particles did not cause aggregation of platelets.

## 4 CONCLUSION AND OUTLOOK

**FIGURE 7:**
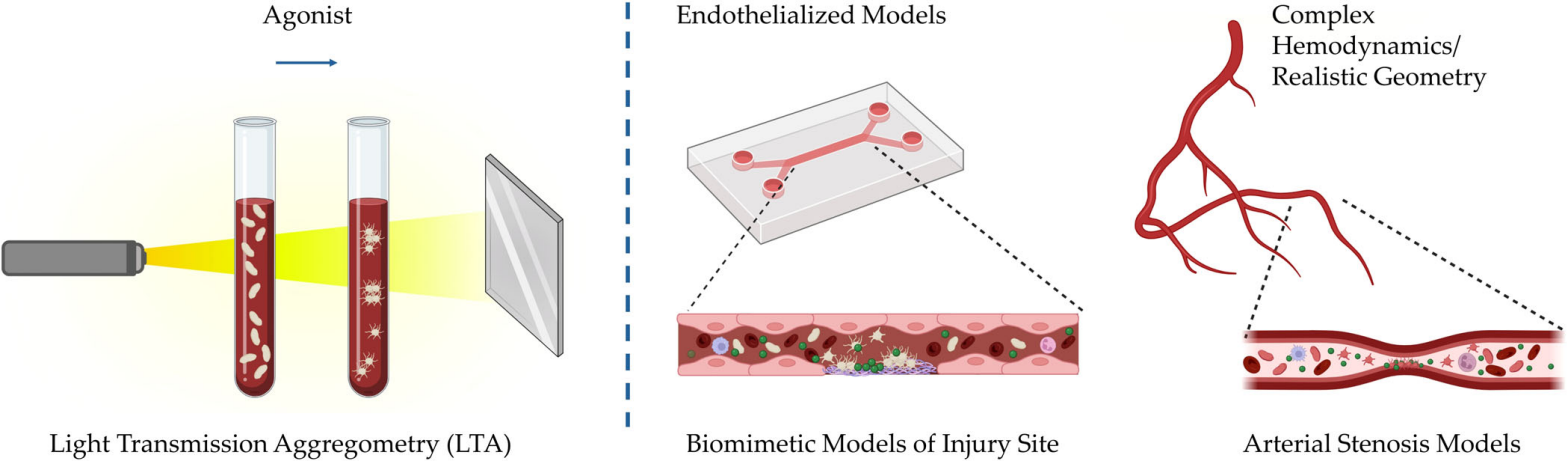
Traditional and advanced methods to study platelet‐NP interactions. Left: Light transmission Aggregometry is the conventional method where upon addition of an agonist platelet aggregation is monitored via Right: Advanced microfluidic models of recapitulating health and disease conditions, incorporating endothelial cells and complex hemodynamic/geometries, can provide valuable information on clinically relevant complex platelet‐NP interactions. Created with BioRender.com.

The effect of NPs on platelets strongly depends on the physicochemical characteristics of the NPs, including their size and surface area. The concentration of the NPs also controls the outcome of these interactions. It is thus critical for all drug delivery NP formulations, which are to be administered via the circulatory system, to consider their complex interaction with platelets to avoid undesired side effects. As presented in our review, there is still a significant gap in knowledge that needs to be bridged in order to enable basic understanding of NP‐platelets interactions. Furthermore, inconsistent trends observed in results from different assays and conditions highlight the necessity for guidance and recommendations on methods and protocols. Plasma proteins play a significant role in platelet‐NP interaction, suggesting the preference for studies using platelet‐rich plasma (PRP) or whole blood over washed platelets to better replicate these interactions. Addressing this knowledge gap requires the development and utilization of high‐throughput standardized methods to generate more data. This may facilitate the application of Artificial Intelligence/Machine Learning algorithms as predictive tools for NP formulations in the future. Additionally, for basic research studies, more complex models incorporating flow and vascular cells are essential to better understand physiological and pathological conditions. As outlined in our review most of the work in the field utilizes mouse models or in vitro assays that do not recapitulate the complex dynamics and interplays that exist in thrombosis or bleeding scenarios in humans.^78^ For example, Light Transmission Aggregometer (LTA) is the traditional gold standard by which platelet function is assessed. However, this approach does not simulate hemodynamic conditions while platelets response and thrombus formation are highly dependent on flow conditions. Therefore, more advanced methodologies need to be considered in evaluating NPs based effects on platelets, see Figure 7. Moreover, human hemodynamic and thrombus dynamic significantly differ from the ones in mice models.^79^ To address these shortcomings, microfluidic models of health and disease conditions have become increasingly attractive. These include stenosis models,^80^, ^81^ bifurcation models,^82^ as well as bleeding models that are currently also being developed and applied.^78^, ^83^, ^84^ Moreover, biomimetic microfluidic models can also incorporate endothelial cells which can also be an important player in understanding NPs interactions with platelet in the vascular system.^85^ Altogether, the interaction of NPs in blood within the human vascular system is still vague and micro‐physiological models can assist us in gaining a better understanding of these processes and in developing NPs to treat vascular disease conditions more effectively.

**FIGURE 8:**
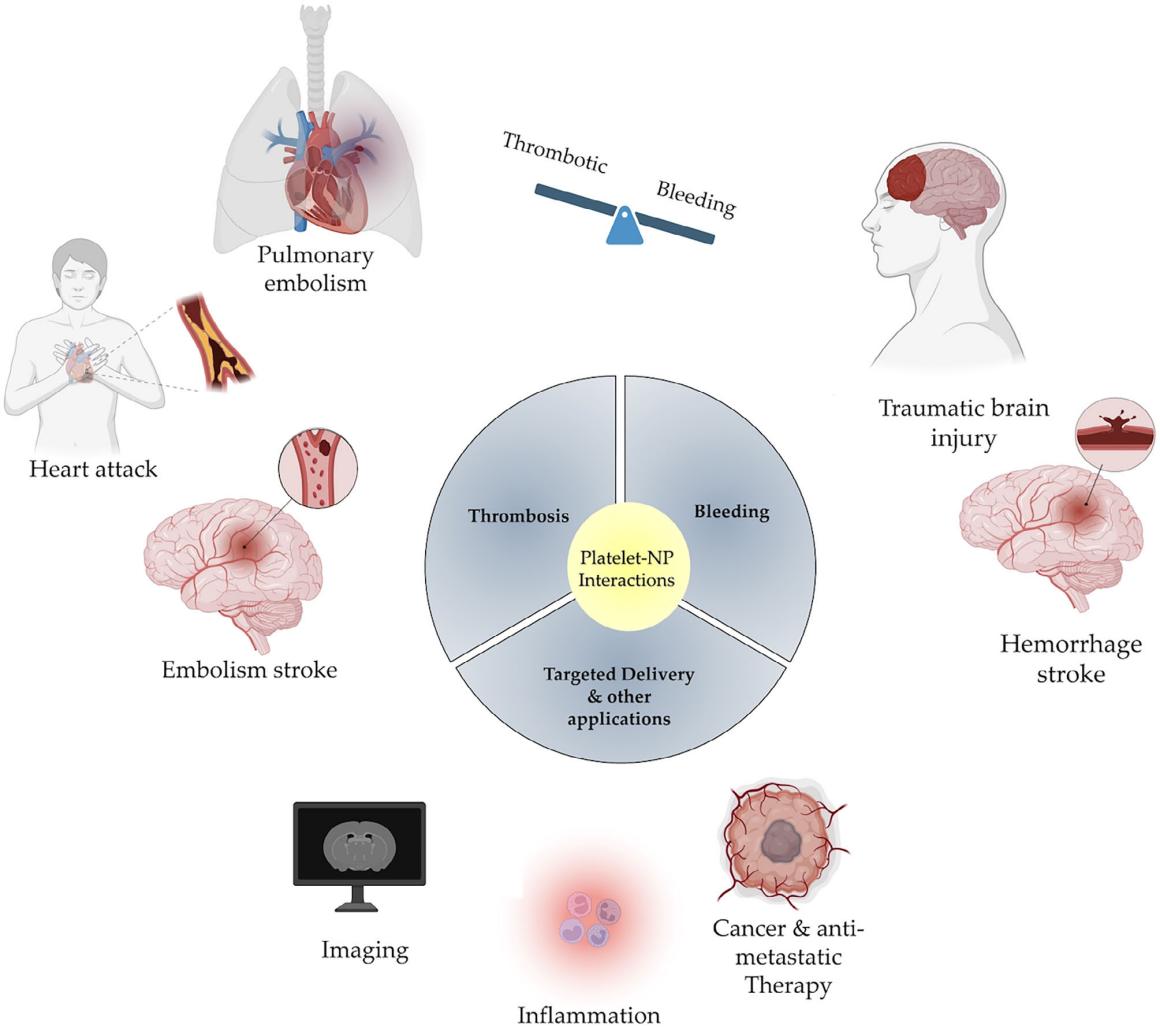
Potential future clinical applications using platelet mimetic NPs and Platelet‐NPs interactions. Platelet mimetic NPs may be used as artificial platelets to treat internal bleedings in different clinical scenarios or alternativity to deliver anti‐clot‐busting agent in thrombo‐embolic conditions. They can also be used to deliver drugs and imaging agents to treat and diagnose cancer, metastasis, inflammation, and other disease conditions. Created with BioRender.com.

Looking towards future clinical applications, the interaction between platelets and NPs holds promise for developing innovative therapeutic approaches that can either impede or augment the crucial roles played by platelets in hemostasis, inflammation, tumor metastasis, wound healing, and host defense. While beyond the scope of this review, it is noteworthy to mention the potential use of functionalized platelet mimetic nanocarriers for targeted drug delivery or as substitutes for artificial platelets, as discussed in reviews^86^, ^87^ (see Figure 8). In the near future, platelet mimetic NPs may be used as artificial platelets that can be transfused to effectively manage internal bleedings in different clinical scenarios ranging from hemorrhagic stroke to traumatic brain injuries to battlefield blast injuries. On the opposite end of the thrombosis‐hemostasis spectrum, platelet mimetic NPs have the potential to target anti‐platelet and clot‐dissolving agents to locally dissolve clots in conditions such as ischemic stroke, myocardial infarction, and pulmonary embolism. Interestingly, stimuli responsive NP formulation, both mechanically and chemically, based on platelet function are also showing promising results in resolving key clinical pathological conditions, such as stroke and hemorrhage.^80^, ^87^, ^88^, ^89^, ^90^, ^91^ Considering the role of platelets in tumor growth and metastasis, the interaction between platelets and NPs can be exploited to develop NPs that interfere with tumor cell metastasis. Additionally, the platelet mimetic NPs can be used for imaging and diagnostics. In summary, while studying NP‐platelet interactions is crucial for developing safe nanomedicines that do not cause adverse clinical effects, it also presents an opportunity to develop new targeted therapeutics for a wide range of clinical scenarios, offering potentially life‐saving treatments.

## AUTHOR CONTRIBUTIONS

**Yathreb Asaad:** Conceptualization; writing – original draft. **Danielle Nemcovsky‐Amar:** Conceptualization; writing – original draft. **Josué Sznitman:** Conceptualization; writing – review and editing. **Pierre Mangin:** Conceptualization; writing – review and editing. **Netanel Korin:** Conceptualization; writing – review and editing.

## CONFLICT OF INTEREST STATEMENT

The authors declare that they have no known competing financial interests or personal relationships that could have appeared to influence the study reported in this article.

### PEER REVIEW

The peer review history for this article is available at https://www.webofscience.com/api/gateway/wos/peer-review/10.1002/btm2.10669.

## Data Availability

The data that support the findings of this study are available from the corresponding author upon reasonable request.
